# Biomarkers in Venous Thrombosis: Diagnostic Potential and Limitations

**DOI:** 10.3390/biology14070800

**Published:** 2025-07-01

**Authors:** Yijin Chang, Jiahao Lu, Changsheng Chen

**Affiliations:** Nantong Laboratory of Development and Diseases, School of Life Sciences, Nantong University, Nantong 226019, China; yijin_chang@163.com (Y.C.); 2409310017@stmail.ntu.edu.cn (J.L.)

**Keywords:** venous thromboembolism, biomarkers, diagnosis, limitations

## Abstract

Venous thromboembolism (VTE), comprising deep vein thrombosis (DVT) and pulmonary embolism (PE), is a life-threatening vascular disorder associated with significant morbidity and mortality. Prompt diagnosis is crucial for preventing fatal complications. Current clinical VTE diagnosis predominantly relies on imaging modalities such as compression ultrasound, computed tomography angiography (CTA), and magnetic resonance imaging (MRI). However, these techniques are resource-intensive, time-consuming, and may expose patients to radiation risks. Consequently, the development of highly sensitive and specific biomarkers is imperative to enhance early detection and guide therapeutic interventions. This review examines established and emerging biomarkers in venous thrombosis, evaluates current challenges, and outlines promising future directions for biomarker research in VTE.

## Simple Summary

Blood clots in veins, known as venous thromboembolism (VTE), can be very dangerous and even fatal if not detected early. Current methods to diagnose these clots, like special ultrasounds and scans, can be time-consuming, expensive, and sometimes expose patients to harmful radiation. Our review looks at various biological markers in the blood that could help detect these clots more quickly and safely. We studied both the markers that are already in use and the new ones being researched. We found that while these markers show promise, they also have limitations, like not being specific enough to VTE. By combining different markers and using new technologies, we hope to develop better diagnostic tools. These improvements could lead to earlier detection of blood clots, better treatment decisions, and ultimately save lives by reducing the burden of this serious condition on patients and healthcare systems.

## 1. Epidemiology of VTE

Venous thromboembolism (VTE) is the third leading cause of vascular death globally, following myocardial infarction (MI) and stroke, and represents a significant public health challenge [1,2]. Its incidence increases markedly after age 45 and is higher in males than in females. Geographically, Western countries report annual incidence rates of 1–2 per mille (‰), compared to less than 1‰ in Asian populations [3]. This disparity may stem from the higher prevalence of factor V Leiden mutations in Caucasian populations versus Asian cohorts [4,5]. Beyond traditional risk factors—such as male sex, smoking, and obesity—prolonged air travel elevates VTE risk by two- to threefold [6]. Hereditary thrombophilia also accounts for a substantial proportion of idiopathic VTE cases [7].

During the COVID-19 pandemic, VTE incidence surged among infected individuals, ranging from 24% to 27% [8]. PE constituted 16.5% of COVID-19-associated VTE cases (range: 11.6–23.4%), while DVT ranged from 7% to 14% [8]. VTE risk correlates with disease severity, with intensive care unit (ICU) patients exhibiting the highest vulnerability. Special populations, including pregnant women and cancer patients, face elevated risks. In pregnancy, DVT predominates, but PE represents the primary cause of VTE-related morbidity and accounts for 10–15% of pregnancy-associated maternal deaths in high-income countries [9,10]. Cancer patients demonstrate a 4- to 7-fold higher VTE incidence than non-cancer individuals, with rates escalating further in cancer-associated VTE [11,12].

Despite its global burden, rising incidence due to population aging and the pandemic, and heightened risks in vulnerable groups, public awareness of VTE remains critically low. This underscores the urgent need for enhanced education, preventive strategies, and timely diagnostic approaches to mitigate VTE-related morbidity and mortality.

## 2. Pathophysiology of VTE

The pathophysiology of VTE is based on Virchow’s triad: hypercoagulability, vascular endothelial injury, and stasis [13]. Hypercoagulability refers to an increased tendency of the blood to clot, which can be caused by various factors such as genetic mutations, cancer, infections, or hormonal changes like those occurring during pregnancy. Vascular endothelial injury involves damage to the inner lining of the blood vessels, which can be due to trauma, inflammation, or conditions like atherosclerosis. Stasis refers to the slowing or stagnation of blood flow, which can occur due to prolonged immobility, heart failure, or other conditions that reduce the velocity of blood in the veins. In the context of VTE, venous thrombi typically form in the valve pockets or dilated sinuses of the veins in the lower limbs. These areas are prone to stasis because the blood flow is slower and more turbulent, creating an environment conducive to clot formation. The thrombi that form are composed of a laminar structure that includes platelets, red blood cells, white blood cells, and fibrin [6,14]. The formation of a thrombus is a dynamic process that involves the activation of ECs. When ECs are activated, they undergo a series of changes that suppress the production of anticoagulant proteins and simultaneously amplify the production of procoagulant factors. This shift in balance promotes further stasis and the growth of the thrombus.

Both acquired and genetic factors can contribute to a state of chronic hypercoagulability. Acquired factors include conditions such as malignancy, where cancer cells can activate coagulation pathways, acute infections and inflammation, which can trigger an immune response that inadvertently promotes clotting, and pregnancy-related increases in estrogen levels, which can enhance the coagulability of blood. Hereditary factors, on the other hand, may involve defects in the body’s natural anticoagulant system. For example, deficiencies in antithrombin III, a protein that helps inhibit clot formation, or protein C, another natural anticoagulant, can significantly increase an individual’s risk of developing VTE [15,16,17]. In addition to these factors, age-related conditions such as diabetes, acute vascular injury, and endothelial dysfunction can also play a role in the development of VTE. These conditions can lead to changes in the blood vessels, such as thickening of the vessel walls, reduced elasticity, and increased adhesiveness of the endothelial surface, all of which can predispose individuals to thrombus formation [18,19].

The development of VTE is driven by the interplay of these genetic and acquired factors. When the balance between the body’s procoagulant and anticoagulant mechanisms is disrupted, it can lead to an excessive tendency for blood to clot. This disruption is a key factor in the pathogenesis of VTE. Understanding the complex mechanisms underlying VTE is crucial for the identification of effective biomarkers. Such biomarkers can aid in the early detection of VTE, allow for a more accurate diagnosis, and guide the development of targeted therapeutic interventions to prevent and treat this potentially life-threatening condition.

## 3. VTE Biomarkers

### 3.1. D-Dimer

D-dimer, a degradation product of cross-linked fibrin resulting from plasmin activity, contains unique linkages between the D and E subunits of fibrin and serves as a marker of coagulation-fibrinolysis imbalance detectable in blood [20]. Since its introduction for VTE diagnosis two decades ago, D-dimer has become a cornerstone in ruling out VTE, particularly in low-to-intermediate probability cases [21]. While healthy individuals exhibit low plasma D-dimer levels, concentrations rise significantly in VTE patients and diminish with symptom duration and anticoagulant therapy [22,23,24]. Clinical measurement of D-dimer can be traced back to the early 1970s, using techniques such as staphylococcal clumping, tanned red-cell hemagglutination inhibition, latex agglutination, and immunodiffusion [25]. With the development of monoclonal antibodies that bind to epitopes specific to D-dimer fragments, which are absent in fibrinogen and non-cross-linked fibrin, the sensitivity and specificity are significantly improved [21,26,27].

As the most widely used VTE biomarker, D-dimer’s high sensitivity (>95%) makes it essential for excluding VTE in emergency or outpatient settings, especially when combined with clinical pre-test probability. Its near-100% negative predictive value (NPV) reduces unnecessary imaging [21,28,29]. However, D-dimer’s low specificity (50–70%) limits its utility, as elevations may occur in cancer, inflammation, cardiovascular disease, infection, trauma, surgery, pregnancy, and aging, causing false positives [28,30,31,32]. The typical reference range for adults is generally below 0.5 µg/mL. However, its levels naturally increase with age, reflecting changes in hemostatic balance and vascular health [33,34]. In high-risk populations—such as pregnant women, cancer patients, and the elderly—diagnostic accuracy declines, necessitating adjunctive imaging. Additionally, variations in detection methods (e.g., ELISA, latex turbidimetry) yield differing sensitivities and specificities, emphasizing the need for method-specific clinical cutoffs [27,28]. Despite these challenges, D-dimer’s high NPV remains critical for VTE exclusion, though its low specificity underscores the importance of combining it with other biomarkers to enhance diagnostic accuracy (Table 1).

### 3.2. Fibrinogen Degradation Products (FDPs)

Fibrinogen degradation products (FDPs), including D-dimer, are fragments generated during fibrinolysis—the enzymatic breakdown of fibrinogen (a clotting factor) or fibrin (the primary component of blood clots) by plasmin [35]. While other FDPs may originate from circulating fibrinogen degradation, D-dimer is uniquely derived from clot breakdown and represents the smallest fibrinolysis-specific degradation product in circulation [20,36]. Although FDPs are less commonly used than D-dimer in VTE diagnosis, their levels rise significantly in acute VTE, making them a potential diagnostic indicator [37]. Studies demonstrate an area under the curve (AUC) of 0.8123 for FDPs in acute VTE diagnosis, with a sensitivity of 34.1% and specificity of 99.5%. However, their diagnostic efficacy is inferior to D-dimer, which achieves an AUC exceeding 0.99 and sensitivity surpassing 95%. In subclinical DVT, FDPs exhibit an AUC of 0.5869 and specificity of 29.3%, yet achieve 93.8% sensitivity and 100% negative predictive value (NPV) for excluding subclinical VTE [37]. These relatively low sensitivity and specificity values limit FDPs’ practicality, particularly in postoperative fibrinolysis settings.

Importantly, FDPs are not specific to VTE, as elevated levels may also occur in non-thrombotic hypercoagulable states such as infection, malignancy, trauma, and disseminated intravascular coagulation (DIC) [38,39,40,41,42]. Moreover, variations in detection methods can significantly impact FDPs’ sensitivity and specificity. While FDPs hold some diagnostic value in acute VTE, their performance in subclinical and postoperative VTE is suboptimal. As a VTE biomarker, FDPs are primarily used in an auxiliary capacity or alongside other tests to complement D-dimer, thereby enhancing diagnostic accuracy (Table 1).

### 3.3. Soluble P-Selectin (sP-Selectin)

Soluble P-selectin (sP-selectin), a cell adhesion molecule from the selectin family, is expressed on activated platelets and ECs and binds to its counter-receptor PSGL-1 on leukocytes and platelets, initiating procoagulant mechanisms [43]. As a potential biomarker for VTE diagnosis, elevated sP-selectin levels reflect platelet and endothelial activation, thrombus formation, and disease progression [44,45,46]. Studies indicate significantly higher sP-selectin levels in VTE patients compared to non-VTE individuals. For example, one study of 100 samples reported VTE patients had mean sP-selectin levels of 75.7 ng/mL versus 53.0 ng/mL in non-VTE patients (*p* < 0.001), achieving 72.7% sensitivity and 78.2% specificity at a 74.8 ng/mL cutoff [47]. When combined with the Wells score, sP-selectin demonstrates enhanced diagnostic performance. Multivariable logistic regression analysis revealed that sP-selectin ≥ 90 ng/mL paired with a Wells score ≥ 2 yielded 33% sensitivity, 95% specificity, and 100% positive predictive value (PPV) for DVT diagnosis, while sP-selectin ≤ 60 ng/mL with a Wells score < 2 achieved 99% sensitivity, 33% specificity, and 96% negative predictive value (NPV) for DVT exclusion [48]. Additionally, elevated plasma sP-selectin independently predicts VTE risk in cancer patients. A multivariable analysis in oncology cohorts found that sP-selectin above the 75th percentile (53.1 ng/mL) was a significant VTE risk factor after adjusting for age, sex, surgery, chemotherapy, and radiotherapy [49]. Despite these findings, sP-selectin’s relatively low sensitivity for VTE diagnosis and susceptibility to inflammation- or malignancy-related elevations may result in false positives, limiting its standalone diagnostic utility (Table 1).

### 3.4. Thrombin–Antithrombin Complex (TAT)

The thrombin–antithrombin complex (TAT), formed when thrombin binds to antithrombin III, serves as a marker of coagulation activation and reflects the functional state of the coagulation system [50]. Unlike D-dimer, which reflects fibrinolytic activity, TAT signals coagulation activation earlier in the pathological process. For instance, TAT levels exhibit significant elevation on postoperative days 3 and 6 in joint arthroplasty patients, underscoring its utility in early VTE detection [51]. As a VTE biomarker in this context, TAT demonstrates superior sensitivity and specificity compared to D-dimer, highlighting its potential for postoperative monitoring [51]. In a study involving 870 patients with malignant tumors (82 with VTE) and 200 healthy controls, the AUC for TAT in VTE diagnosis was 0.875, with a sensitivity of 85.6% and a specificity of 75.4% at a cutoff value of 30.76 µg/L, showing better diagnostic accuracy than D-dimer and FDPs [52]. Following recombinant factor VII (rFVIIa) administration, TAT levels transiently increase and remain elevated in VTE patients relative to healthy controls. Furthermore, TAT correlates with the fibrinolytic marker plasmin–alpha2–antiplasmin complex (PAP) in VTE patients but not in healthy individuals, reinforcing its role in VTE risk prediction models [53].

However, TAT has notable limitations as a VTE diagnostic tool. Its specificity is compromised by activation of coagulation in inflammatory and infectious conditions, rendering it non-specific to VTE. In lung cancer-associated VTE, TAT specificity is as low as 48.1%, with false positives occurring due to other coagulation-activating states such as inflammation or trauma [54]. In primary care settings, TAT does not significantly enhance the diagnostic performance of D-dimer for VTE. Additionally, TAT’s short half-life and susceptibility to proteolytic degradation necessitate timely detection post-coagulation activation, as its levels rise rapidly but diminish soon after VTE onset [55]. Genetic factors also influence TAT measurements; for example, asymptomatic FV Leiden carriers exhibit no significant difference in TAT response compared to symptomatic carriers [53]. TAT levels correlate with clinical status, yet they fail to distinguish between pathological coagulation activation in VTE and other conditions, as evidenced by comparable TAT levels in VTE patients and controls during fat tolerance tests [56].

In summary, while TAT is a key biomarker reflecting coagulation activation in VTE, its diagnostic utility is limited by low specificity and genetic influences. Combining TAT with other biomarkers like D-dimer may improve diagnostic efficacy, though its role in low-risk populations remains underexplored and warrants further investigation (Table 1).

### 3.5. Plasmin–Alpha2–Antiplasmin Complex (PAP)

The plasmin–antiplasmin system plays a pivotal role in blood coagulation and fibrinolysis. Plasmin and α2-antiplasmin (α2-AP) are key regulators of fibrin polymer dissolution into soluble fragments. The plasmin–antiplasmin complex (PAP) forms through a 1:1 binding of plasmin to α2-AP [57]. During thrombus formation, the fibrinolytic system is activated, converting plasminogen to plasmin, which degrades fibrin, a primary component of thrombi. Free plasmin rapidly combines with α2-AP to form stable PAP. Elevated PAP levels indicate activated fibrinolysis and thrombus formation, making it a useful marker for early VTE screening [58,59,60]. In a study of gynecological malignancy patients, PAP demonstrated an area under the curve (AUC) of 0.95 for preoperative VTE diagnosis and 0.941 postoperatively [61]. As PAP directly reflects plasmin activity and rises earlier in thrombus formation than D-dimer, it is particularly suitable for early VTE detection. Additionally, combining PAP with other biomarkers has been shown to enhance sensitivity and specificity in malignant tumor patients with VTE, outperforming D-dimer alone [52]. However, a study by Folsom et al. found no significant association between fibrinolytic variables and VTE after adjusting for age or multiple VTE risk factors [62]. Current research on PAP as a VTE biomarker primarily focuses on specialized populations, such as postoperative oncology patients. Its effectiveness in broader populations and generalizability requires further validation. Some studies are limited by small sample sizes and low statistical power. Despite these limitations, PAP exhibits high detection efficiency and accuracy in malignant tumor-related VTE, particularly in postoperative monitoring. Combining PAP with multiple markers may further improve diagnostic efficacy (Table 1).

### 3.6. Prothrombin Fragment F1+2 (F1+2)

The conversion of prothrombin to thrombin represents a critical step in blood coagulation. Prothrombin fragment F1+2 (F1+2), an activation peptide released during thrombin formation, serves as a marker of coagulation activation [63]. Elevated F1+2 levels are observed in patients with VTE and MI [64]. Ota et al. have found that F1+2 was elevated in more than 50% of patients with thrombosis [65], and it was considered to be useful for the diagnosis of thrombosis, which demonstrates diagnostic utility comparable to D-dimer and the TAT complex [66]. In a study of 252 patients undergoing total knee arthroplasty (TKA), the AUC for F1+2 was 0.797, with a sensitivity of 74.6% and a specificity of 78.3 at the optimal cutoff value of 11.1 ng/mL. This indicates that F1+2 has relatively high sensitivity in detecting VTE in this population [67]. During pregnancy, physiological changes induce a hypercoagulable state; F1+2 levels rise significantly with gestational age, positioning F1+2 as a potential indicator of pregnancy-related hypercoagulation [68]. Furthermore, F1+2 aids in detecting coagulation abnormalities in cancer patients and predicting cancer-associated VTE [69,70,71]. However, F1+2 has limitations in VTE diagnosis. Its levels are influenced by various biological factors and may be elevated in conditions such as disseminated intravascular coagulation (DIC), sepsis, trauma, arterial disease, cancer, and postoperative states, reducing its specificity [69,72,73,74,75,76] (Table 1).

### 3.7. Microparticles (MPs)

Microparticles (MPs), also known as microvesicles, are small membrane fragments (0.1–1 μm in diameter) released by activated, damaged, or apoptotic cells [77,78]. These vesicles carry bioactive molecules and play diverse roles in physiological and pathophysiological processes [79,80]. Research has found that MPs are markedly elevated in patients with VTE and have the potential to serve as biomarkers for VTE [81,82]. Peripheral blood MP detection aids early VTE diagnosis, particularly in high-risk groups such as orthopedic surgery patients or those with cancer [83,84,85]. For instance, annexin V− and tissue factor+ MPs are effective early postoperative VTE biomarkers in joint arthroplasty [83]. In a study involving 102 PE patients, 102 healthy controls, and 40 patients suspected of PE, MP levels were significantly higher in PE patients (609.10/µL) compared to healthy controls (230.60/µL) and suspected PE patients (166.70/µL; *p* < 0.01). ROC curve analysis showed that PMPs had a sensitivity of 93.14% and a specificity of 51.96% for PE diagnosis at a cutoff value of 236.97/µL (AUC area 0.822). The accuracy (72.06%) of MPs in the diagnosis of PE was similar to that of D-dimer (*p* > 0.05) [86]. However, MPs are influenced by conditions like inflammation and cancer, and elevated levels alone cannot confirm VTE; thus, clinical symptoms and other diagnostic results are essential for comprehensive assessment. Furthermore, studies indicate that P-selectin or D-dimer, alone or combined, offer no superior sensitivity or specificity compared to MPs [87,88]. Additionally, MP research is hindered by detection method limitations. Techniques such as ELISA, flow cytometry, and electron microscopy vary in technical demands and procedures [84,87,89]. Variations in methods and equipment can yield inconsistent results, affecting MP biomarker accuracy and reproducibility (Table 1).

### 3.8. C-Reactive Protein (CRP)

C-reactive protein (CRP), an acute-phase reactant produced by the liver, is rapidly synthesized in response to infection and inflammation [90]. Emerging evidence indicates that CRP is not merely an inflammatory marker but also possesses pro-inflammatory and pro-thrombotic properties through its destabilized isoforms [91,92]. Given inflammation’s role in driving VTE, the association between CRP and VTE risk is of significant interest [93]. Kunutsor et al. conducted a meta-analysis of nine prospective cohort studies, revealing a link between elevated CRP levels and increased VTE risk [94]. A population-based case-crossover study further confirmed this association [95]. In acute settings, individuals with acute DVT exhibit higher circulating CRP levels compared to those without DVT [96,97]. However, elevated CRP lacks specificity for VTE, as it is also observed in various inflammatory conditions, including infections, autoimmune diseases, and tissue injuries. Consequently, CRP alone cannot definitively diagnose VTE or distinguish it from other inflammatory pathologies (Table 1).

### 3.9. Homocysteine

Homocysteine, an amino acid synthesized during methionine metabolism through vitamin B6- and B12-dependent pathways, is implicated in several disease processes [98]. Elevated homocysteine levels are associated with increased risks of cardiovascular disease, stroke, neurodegenerative disorders (e.g., dementia, Alzheimer’s disease), birth defects, complicated pregnancies, and bone fractures [99]. Mechanistically, hyperhomocysteinemia may promote thrombosis through multiple pathways, including increased tissue factor expression, impaired anticoagulant processes, heightened platelet reactivity, augmented thrombin generation, enhanced factor V activity, reduced fibrinolytic potential, and vascular endothelial injury [100]. However, the role of homocysteine as a risk factor and biomarker for VTE remains controversial. A meta-analysis of case-control studies reported that each 5 μmol/L increase in plasma homocysteine is associated with a higher VTE risk, suggesting a potential role in VTE pathogenesis [101]. Similar findings emerged from the FIELD study, where a 5 μmol/L elevation in baseline homocysteine corresponded to a 19% increased VTE risk [102]. Ekim et al. also identified hyperhomocysteinemia as a risk factor for DVT in women aged over 40 [103]. Conversely, a case-control study by Ducros et al. found no statistically significant link between hyperhomocysteinemia and VTE, questioning its utility as a biomarker [104]. Additionally, elevated homocysteine levels may arise from renal disease, vitamin deficiencies, or genetic disorders, limiting its specificity for VTE [105,106]. These factors, combined with the modest association observed in studies, restrict homocysteine’s clinical utility as a VTE biomarker (Table 1).

### 3.10. Endocan

Endocan, also known as endothelial-specific molecule 1 (ESM-1), is a proteoglycan primarily expressed in ECs and regulated by cytokines such as tumor necrosis factor-α (TNF-α), interleukin (IL)-1, and microbial lipopolysaccharide, as well as proangiogenic factors like vascular endothelial growth factor (VEGF) [107,108,109]. Research indicates that endocan levels correlate positively with endothelial dysfunction severity and serve as a biomarker for this condition [110,111]. Notably, serum endocan levels are significantly elevated in patients with PE and are associated with disease severity [112,113]. Moreover, plasma endocan shows potential as a biomarker for predicting thrombotic events in COVID-19 patients [114]. Conversely, Mosevoll et al. reported no significant differences in plasma endocan levels among patients with DVT, healthy controls, and individuals suspected of having DVT [115]. Additionally, endocan levels may rise in other conditions, including infections and malignancies, which could compromise its specificity for VTE diagnosis [116]. Given these conflicting findings and the potential for nonspecific elevation, further investigation is necessary to establish endocan’s efficacy and reliability as a VTE biomarker (Table 1).

**Table 1:** Biomarkers in venous thromboembolism (VTE) diagnosis.

Biomarkers	Sensitivity (95% CI)	Specificity (95% CI)	Advantages	Limitations	Ref.
D-dimer	>95%	50–70%	high NPV for VTE exclusion; good AUC performance	relatively low specificity	[22,23,24,28,117]
Fibrinogen degradation products (FDPs)	>90%	varied (30–90%) in different populations	high specificity for acute VTE; high NPV for subclinical VTE exclusion	low diagnostic accuracy in subclinical VTE	[37]
Soluble P-selectin (sP-selectin)	>70%	>90%	high NPV for DVT exclusion	moderate diagnostic performance	[47,48,49]
Thrombin–antithrombin complex (TAT)	>85%	>75%	high sensitivity; early signals	specificity is compromised under inflammatory or infectious conditions	[51,52,53]
Plasmin–alpha2–antiplasmin complex (PAP)	>70%	>80%	high specificity and specificity for VTE diagnosis in malignant tumor patients	applications confined to specialized populations	[52,60,61]
Prothrombin fragment F1+2 (F1+2)	>70%	>70%	highly sensitive to thrombus formation; early signals of hypercoagulability	elevated in various conditions; limited diagnostic accuracy in specific populations	[67,68,69]
Microparticles (MPs)	>90%	approx. 50%	high effectiveness in PE diagnosis	influenced by other disease conditions; measurement limitations and variations in results	[82,84,86,88,89]
C-reactive protein (CRP)	undefined	undefined	early diagnosis of acute-phase in VTE	influenced by various inflammatory conditions and not specific to VTE	[94,95,97]
Homocysteine	undefined	undefined	strong association with unprovoked VTE	not specific to VTE and elevated in various disease conditions	[101,102,103,104]
Endocan	>80%	50–75%	early VTE detection; strong association with PE severity	low specificity; lack of standardized cutoff values	[112,113,114]

## 4. Challenges and Future Directions for VTE Biomarkers

VTE, being one of the top three global causes of death, necessitates effective biomarkers for early detection, accurate diagnosis, and reliable prognosis. Current biomarkers, however, have significant limitations. D-dimer, the most widely used biomarker, suffers from low specificity, leading to false positives in conditions such as cancer, inflammation, arterial diseases, trauma/surgery, pregnancy, and aging [28,30,31,32]. Additionally, variations in D-dimer detection methods (e.g., ELISA, latex turbidimetry) and laboratory-specific cutoff values markedly affect its diagnostic accuracy, necessitating standardized protocols. The TAT complex has a short half-life and is unstable, requiring timely detection post-coagulation activation. Its specificity is also low, influenced by genetic factors [55], as seen in lung cancer patients where TAT specificity is only 48.1% [54]. Traditional biomarkers universally face challenges in sensitivity and specificity due to interference from physiological and pathological states (e.g., aging, postoperative status) and insufficient sensitivity for subclinical or early VTE.

Diagnosing VTE with a single biomarker is insufficient. Combining complementary biomarkers can significantly enhance diagnostic efficacy. Multiplex models achieve very high specificity, outperforming traditional D-dimer detection and reducing false positives. In hospitalized patients with high interference factors (e.g., inflammation, cancer), multiplex biomarkers maintain high specificity, minimizing non-VTE interference (e.g., postoperative reactions, chronic inflammation). However, the economic feasibility and clinical applicability of biomarker combinations require further refinement and validation through clinical trials. Customized biomarker combinations for different populations (e.g., cancer patients, elderly) are also needed. With advancements in artificial intelligence (AI), optimizing multiplex biomarker combinations using machine learning is a promising research direction. Accelerating the development of rapid detection chip technologies for simultaneous multiplex analysis will promote the comprehensive application of multiplex biomarkers.

The performance of VTE biomarkers can vary significantly across different populations. Special populations, including cancer patients, pregnant women, the elderly, and bedridden patients, require personalized approaches to VTE diagnosis and treatment. As the global cancer burden increases, research into cancer-associated VTE is gaining momentum. However, personalized VTE risk prediction, diagnosis, and treatment for these special populations face several challenges, such as the non-adaptability of universal VTE biomarker cutoffs to individual differences. To address this, age-specific D-dimer cutoffs have been established to enhance the exclusion of pulmonary embolism (PE), substantially reducing false positives while maintaining high sensitivity. Genetic risk stratification is another tool that can be employed. Research indicates that a polygenic risk score (PRS) model incorporating potential single-nucleotide polymorphisms (SNPs) for VTE in the Chinese population achieves a relatively high area under the curve (AUC), outperforming traditional genome-wide association studies (GWAS) models [118]. Future research should focus on developing personalized risk prediction models to improve detection accuracy and efficacy.

Biomarkers are increasingly important for VTE diagnosis and management. Despite current challenges, advancing research and technology may lead to the emergence of more effective biomarkers, improving diagnostic and treatment strategies for VTE patients.

## 5. Conclusions

Venous thromboembolism (VTE) is a life-threatening vascular disorder associated with significant morbidity and mortality. Timely diagnosis is critical for preventing fatal complications. This review provides a comprehensive overview of the diagnostic potential and limitations of established and emerging VTE biomarkers. It examines various biomarkers, including D-dimer, fibrinogen degradation products (FDPs), soluble P-selectin (sP-selectin), thrombin–antithrombin complex (TAT), plasmin–alpha2–antiplasmin complex (PAP), prothrombin fragment F1+2 (F1+2), microparticles (MPs), C-reactive protein (CRP), homocysteine, and endocan. The review also addresses challenges in VTE biomarker research, such as issues with specificity and genetic influences. Furthermore, it proposes future directions, including the use of multiplex models optimized with machine learning and the development of rapid detection chip technologies. The insights and findings presented may contribute to advancements in VTE diagnosis and management, potentially improving patient outcomes and reducing the burden of this life-threatening condition. Despite current challenges, progress in research and technology may lead to more effective biomarkers, enhancing diagnostic and treatment strategies for VTE patients.

## Data Availability

No new data were created.
